# Treated Livestock Wastewater Irrigation Is Safe for Maize (*Zea mays*) and Soybean (*Glycine max*) Intercropping System Considering Heavy Metals Migration in Soil–Plant System

**DOI:** 10.3390/ijerph20043345

**Published:** 2023-02-14

**Authors:** Rakhwe Kama, Yuan Liu, Jibin Song, Abdoul Kader Mounkaila Hamani, Shouqiang Zhao, Siyi Li, Sekouna Diatta, Fengxia Yang, Zhongyang Li

**Affiliations:** 1 Institute of Farmland Irrigation of CAAS, Xinxiang 453002, China; 2 Laboratory of Ecology, Faculty of Sciences and Technology, Cheikh Anta University of Dakar, Dakar 50005, Senegal; 3 Agro-Environmental Protection Institute, Ministry of Agriculture and Rural Affairs, Tianjin 300191, China; 4 National Research and Observation Station of Shangqiu Agro-Ecology System, Shangqiu 476000, China

**Keywords:** treated wastewater, maize, soybean, heavy metals, intercropping system

## Abstract

Water deficit is a major problem affecting crop production worldwide. The use of treated wastewater in irrigation systems improves soil health and enhances crop growth and productivity. However, it has been characterized as a source of heavy metals. The unknown is how heavy metals’ movements would be impacted under an intercropping system when irrigated with treated wastewater. Understanding the dynamic of heavy metals in soil–plant systems is essential for environmental risk assessment and sustainable agriculture. A greenhouse pot experiment was conducted to explore the effects of treated wastewater irrigation on plant growth, soil chemical properties, and the movements of Zn, Cu, Pb, and Cd from soil to plants in monoculture and intercropping systems. Maize and soybean were selected as the test crops and groundwater and treated livestock wastewater as the water sources. This study found that treated wastewater irrigation and intercropping systems synergically increased the soil nutrient content and crop growth. The concentrations of Zn, Pb, and Cd were significantly higher in leaves compared to other plant parts contrastingly to Cu, which was higher in roots. In addition, treated wastewater irrigation increased grain nutrient content in mono- and intercropping systems while the concentration of heavy metals was in the acceptable range for human consumption. The enrichment degree of Cu and Pb due to treated livestock wastewater irrigation relative to groundwater irrigation was higher in uncultivated soil compared with cultivated soil. This study showed that the intercropping system facilitated heavy metals’ transfer from soil to plant except for Cd. These findings provide guidelines for a safe utilization of treated wastewater in agricultural systems and to reduce freshwater use pressure.

## 1. Introduction

The agricultural system is facing many challenges, including water scarcity, soil pollution, and the lack of cultivable land [1,2,3]. Water scarcity has been and is still a crucial problem for agricultural production worldwide especially in arid and semi-arid regions [4,5]. In addition, water deficit constitutes one of the major problems affecting worldwide crop production [6,7,8,9,10,11]. Thus, the necessity to provide solutions to acquire abundant water resources to improve agricultural production without depriving future generations. Wastewater reuse is considered as a suitable alternative for water deficit in agricultural systems because of its huge volume of discharge. For instance, China’s livestock and poultry manure production in 2020 was 3.05 billion tons [12]. However, efficient treatment is required to determine its impact on the current soil fertility level and continuously monitor the content of salt and heavy metals to assess the risks of soil contamination.

Although wastewater reuse in irrigation systems enhances crop growth and increases soil fertility, it is also considered as a source of heavy metals and organic pollutants [13]. Simply treated wastewater irrigation may increase heavy metals pollution, which is a crucial environmental and health problem because of adverse impacts on the soil and food chain. For instance, organic pollutants originated from wastewater are discharged simultaneously during wastewater irrigation which disturbs biological function and their accumulation causes serious diseases [14,15]. Previous studies showed that the non-degradable heavy metals can be bioaccumulated with relatively high contents in the food chain when consumable crops are grown in heavy metals-contaminated soil or irrigated with roughly treated wastewater [16,17,18,19]. It is clear that consumable crops contamination constitutes a vital pathway of heavy metals exposure. Therefore, it is crucial to understand and assess heavy metals’ transfer in plant–soil system under wastewater irrigation to provide solutions for a safe utilization of treated wastewater in irrigation systems without compromising human and soil health.

Sustainable agriculture is a key element in increasing food production and improving soil health. It is currently encouraged in developing countries including densely populated countries such as China and India where more tasks are required to increase their production [20]. In addition, the current worldwide cultivable land continues to decrease with the augmentation of the global population and industrialization, while global demand for food is increasing. Therefore, alternatives need to be found to face these challenges related to water scarcity, soil pollution, lack of cultivable lands, and decrease in crop production. The intercropping system, an efficient system for environmental resources utilization, is a famous and widely used concept which permits farmers to make full use of the available cultivable land and increase their production [21,22,23]. Intercropping is adopted in many parts of the world, has been a common cropping strategy in China for centuries, and can be considered as a way towards sustainability [24]. In addition, the intercropping system is also an economical and effective cropping pattern, with great advantage over sole cropping in terms of pollution reduction [25]. However, many uncertainties still exist on the effects of treated wastewater irrigation on heavy metals migration under the intercropping system. Since ecologically and environmentally friendly farming methods need to be adopted, understanding heavy metals mobility in treated livestock wastewater-irrigated soil under the intercropping system could be a significant step forwards in providing alternatives for the safe utilization of treated wastewater in agricultural systems.

The hypothesis of this study is that the intercropping system would enhance heavy metals’ transfer efficiency in soil–plant systems. A greenhouse experiment was conducted to comprehensively (i) investigate the effects of treated livestock wastewater irrigation on plant growth, (ii) examine the effects of wastewater irrigation on soil chemical properties under the intercropping system, and (iii) determine the effects of the intercropping system on Zn, Cu, Pb, and Cd migration in the soil–plant system in wastewater irrigation.

## 2. Materials and Methods

### 2.1. Site Description and Experimental Design

A pot experiment was conducted at the Agricultural Water and Soil Environmental Field Science Observation Research Station of Chinese Academy of Agricultural Sciences at Xinxiang (35.27° N, 113.93° E). The average temperature and relative humidity inside the greenhouse during the cultivation period from April to July were, respectively, 33.35 °C and 34%. The pots (38 cm upper diameter and 38.6 cm height) were filled with 30 kg of air-dried sandy loam soil and mixed thoroughly with 75 g of compound fertilizer (N-P_2_O_5_-K_2_O 15:15:15, nutrient ≥45%). The basic chemical characteristics of the used soil, groundwater, and livestock wastewater extracted using standards procedures [26] are shown in Table 1.

The experimental design included two water treatments: treated livestock wastewater diluted 40 times following the standard for water irrigation [27] before application and groundwater and four planting patterns: no planting (CK), monocultured maize (MM), monocultured soybean (MS), and intercropped maize/soybean (IMS) (Table 2). The parent material of soil is Yellow River sediment Potassium oxide (K_2_O) 3.61% Free iron oxide (Fe_2_O_3_) 4.87% CaCO_3_ 9.98% (ACTA PEDOLOGICA SINICA). The livestock wastewater here was the biogas slurry taken from the fermented anaerobic fermentation tank of the Class I intensive pig farm in Xinxiang Shengda Animal Husbandry Co. The groundwater was the shallow groundwater pumped from the experimental site. Seeds were directly sown in soil-filled pots; the number of maize and soybean sown seeds were, respectively, 4 and 8 in monoculture and 3 and 6 in intercropping system. Uniform seedlings were selected after two weeks to reduce the number of maize and soybean to, respectively, 2 and 4 plants per pot in monoculture and 1:2 in intercropping system. Each treatment was replicated 6 times. During the cultivation period, pots were kept under natural light, and crops were irrigated every 2–3 days to maintain soil moisture at 60–70% of the field water holding capacity.

### 2.2. Soil and Plant Sampling

The soil/plant sampling was conducted at maize maturity stage. Monocropped maize and soybean plants were vigilantly taken out from the pots, and the soil was collected from the roots after shaking was considered as rhizosphere soil. For intercropping system, soil samples were collected from the central points where two plants’ roots intersected. The collected soil samples were sealed in polythene bags then sent to a commercial laboratory for chemical analysis. The plants were divided into roots, stems, and leaves. Root samples were washed with tap water to remove dust sand and then washed with deionized water before oven-dried with shoots at 65 °C for 48 h and kept in well-sealed plastic bags until further analysis.

### 2.3. Soil Analysis

Soil chemical analysis was conducted at Key Laboratory of High-efficient and safe Utilization of Agricultural Water Resources, Institute of Farmland Irrigation of CAAS, P.R. China. Sample suspension with soil to water ratio of 1:2.5 was used to determine soil pH and EC. The potassium dichromate oxidation method [28] was used to determine soil OM. Total nitrogen (TN) was determined by the Kjeldahl method [29,30]. Soil TP, water-soluble Na^+^, and water-soluble K^+^ were determined using standards methods [30]. For the heavy metals’ determination, 0.5 g of sieved soil was digested with 15 mL mixture of HNO_3_, H_2_SO_4_, and HClO_4_ in 5:1:1 ratio at 80 °C until a transparent solution was obtained (USEPA Method: 3005A). Mixture was then cooled before filtration with a filter paper and volume was raised to 50 mL with distilled water. The atomic absorption spectrophotometer (AAS PE900H) was then used to determine the concentration of Zn, Cu, Pb, and Cd.

### 2.4. Plant Analysis

Plant height, stem diameter, leaf area, leaf weight ratio, leaf chlorophyll content, and nitrogen level were determined at the end of the experiment. The root, stem leaf, and grain subsamples of plants were ground to pass a 0.5 mm sieve and analyzed to determine TN and heavy metals concentrations. Plant chemical analysis was also conducted at the Key Laboratory as mentioned above. TN was determined by the Kjeldahl method [29] followed by steam distillation. For determination of Zn, Cu, Pb, and Cd in plants, the methods used by [31] were followed.

### 2.5. Statistical Analysis

#### 2.5.1. The Enrichment Factor (EF) and Translocation Factor (TF)

EF and TF were calculated for Cd, Pb, Znm and Cu. The EF was calculated to determine the degree of heavy metal migration and accumulation in soil or plants irrigated with treated livestock wastewater compared to that with groundwater. The enrichment factor was determined using the following equation [32]:

(1) $$EF=\frac{concentration\;of\;heavy\;metals\;in\;soil\;or\;plant\;in\;ww\;irrigated\;soil}{\mathrm{concentration}\;\mathrm{of}\;\mathrm{heavy}\;\mathrm{metals}\;\mathrm{in}\;\mathrm{gw}\;\mathrm{irrigated}\;\mathrm{soil}}$$

where gw: groundwater, and ww: wastewater.

The TF was calculated to determine the relative translocation of metals from roots to other parts (e.g., shoots) of the plants [33].

(2) $$TF=\frac{\mathrm{Concentration}\;\mathrm{of}\;\mathrm{metal}\;\mathrm{in}\;\mathrm{plant}\;\mathrm{shoot}\;}{\mathrm{Concentration}\;\mathrm{of}\;\mathrm{metal}\;\mathrm{in}\;\mathrm{corresponding}\;\mathrm{root}\;}$$

#### 2.5.2. Bioaccumulation Factor

The ability of maize and soybean to accumulate heavy metals from soil was estimated using the bioaccumulation factor (BAF) that was calculated as the ratio of the metal concentration in plant root to that in soil [34]:

(3) $$BAF=\frac{\mathrm{Concentration}\;\mathrm{of}\;\mathrm{metal}\;\mathrm{in}\;\mathrm{plant}\;\mathrm{root}\;}{\mathrm{Concentration}\;\mathrm{of}\;\mathrm{metal}\;\mathrm{in}\;\mathrm{corresponding}\;\mathrm{soil}\;}$$

#### 2.5.3. The Transfer Coefficient (TC)

TC of Zn, Cu, Pb, and Cd was the ratio between the concentration of a metal in plants by the concentration of that metal in the soil. High TC reflects relatively poor retention in soil or greater efficiency of plants to absorb metal, and low TC reflects the strong sorption of metal to the soil colloid [35].

(4) $$TC=\frac{\mathrm{Concentration}\;\mathrm{of}\;\mathrm{metal}\;\mathrm{in}\;\mathrm{plants}\;}{\mathrm{Concentration}\;\mathrm{of}\;\mathrm{metal}\;\mathrm{in}\;\mathrm{corresponding}\;\mathrm{soil}\;}$$

The data were analyzed using SPSS 20.0 and presented as mean ± standard error. Statistical differences between growth characteristics and the concentrations of Pb, Cd, Cu, and Zn in plants and soils were determined by Tukey’s Multiple Range Test at *p* < 0.05 level. Comparisons between the treatments were made by using evaluation statements in two-way ANOVA, and multiple comparisons among treatments were performed using the Tukey–Kramer pattern. Treatment and interaction effects were considered significant when *p* < 0.05. Origin Pro version 2021b software was used for figure construction.

## 3. Results

### 3.1. Effects of Treated Wastewater Irrigation on Plant Growth under Different Planting Patterns

Maize and soybean growth parameters were differently affected by water treatments and the type of culture. Maize and soybean plant height and stem diameter were greater in treated wastewater irrigation under both cropping systems (Table 3a,b). Maize shoot dry weight (SDW) was greater in groundwater irrigation and under the intercropping system contrastingly to soybean in which the SDW was greater in treated wastewater irrigation and monoculture. The maize and soybean leaf weight ratio (LWR) was increased under the intercropping system in treated wastewater irrigation compared with monoculture. Maize leaf area with treated livestock wastewater irrigation increased under the intercropping system in comparison with monoculture (Table 3a).

Soybean leaf area was greater in monoculture in wastewater treatment compared with the intercropping system (Table 3b). However, intercropped soybean leaf area was higher than monocropped soybean in groundwater irrigation. The results showed that treated livestock wastewater has a significant influence on the crop’s leaf area (Table 3a,b).

A two-way ANOVA was performed to determine whether water treatments and planting patterns had significant effects on crop growth. Water treatment had significant effects on plant growth parameters except on plant height for both crops and soybean SDW. The planting patterns effect was significant for both crops’ growth parameters except for maize plant height and stem diameter. Interactions between water treatments and planting patterns significantly affected maize and soybean growth parameters except soybean stem diameter and SDW.

**Figure 1:**
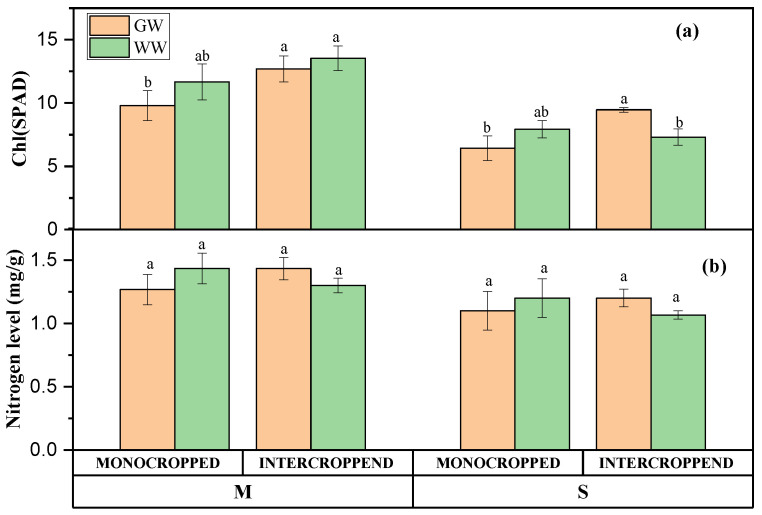
Plant leaf chlorophyll content (**a**) and nitrogen level (**b**) under different water treatments and planting patterns. Note: M: maize; S: soybean; GW: groundwater; WW: wastewater. All data are represented as means ± standard errors (n = 6). Different letters indicate statistically significant differences between treatments at *p* < 0.05.

The one-way ANOVA analysis of maize/soybean leaf chlorophyll content and nitrogen level showed significant differences depending on water treatments and planting patterns (Figure 1). Maize leaf chlorophyll content was greater in treated wastewater irrigation under the intercropping system compared with groundwater and monoculture. This study showed that the intercropping system increased maize leaf chlorophyll content. Maize leaf nitrogen level was higher in groundwater irrigation under the intercropping system and in wastewater irrigation plus the monoculture system. Interestingly, soybean leaf chlorophyll content and nitrogen level were higher in groundwater irrigation under the intercropping system but greater in wastewater irrigation in monoculture. The results showed that water treatments and planting patterns had significant effects on leaf chlorophyll content and nitrogen level.

### 3.2. Effects of Treated Wastewater Irrigation and Intercropping System on Crops Yield

The results showed that treated livestock wastewater irrigation increased maize yield under the intercropping system while maize yield was higher under the monocropping system in groundwater-irrigated soil (Table 4a). However, optimal maize yield was observed in treated wastewater irrigation under the intercropping system. Interestingly, soybean yield was significantly increased by treated wastewater irrigation in monoculture compared with the intercropping system (Table 4b). Soybean yield was greater in treated wastewater irrigation under the monocropping system compared with groundwater. Contrastingly to the monocropping system, soybean yield was higher under groundwater irrigation compared with treated wastewater under the intercropping system (Table 4b).

### 3.3. Effects of Water Treatments and Planting Patterns on Soil Properties

The concentrations of TN, TP, water-soluble Na^+^, and water-soluble K^+^ increased in treated wastewater-irrigated soil with higher values observed in uncultivated soil. The same situation was observed for soil EC. No significant difference was observed on soil pH and OM regardless of water treatment or planting patterns. This study showed that soil chemical properties were significantly affected by the planting patterns (Table 5).

### 3.4. Heavy Metals Concentration in Soil and Enrichment Factor

**Figure 2:**
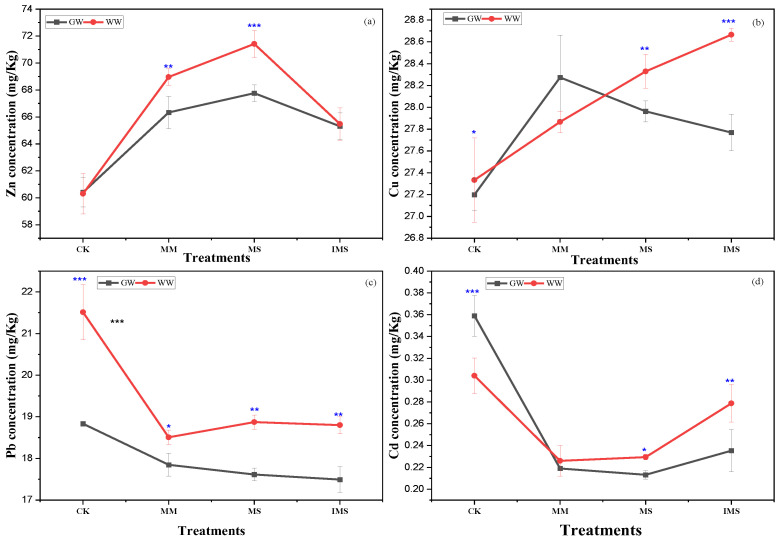
Concentrations of Zn (**a**), Cu (**b**), Pb (**c**), and Cd (**d**) in soil under monoculture and intercropping systems. Note: GW: groundwater; WW: wastewater; CK: uncultivated soil; MM: maize monocultured soil; MS: soybean monocultured soil; IMS: maize/soybean intercropped soil. All data are represented as means ± standard errors (n = 6) (* p < 0.05, ** *p* < 0.001, *** *p* < 0.0001).

Significant differences were observed on Zn concentration depending on water treatments, with higher concentration observed in treated wastewater irrigation and, respectively, under monocultured soybean and maize (Figure 2a). The Cu concentration in soil was highly impacted by water treatments and planting patterns. Higher concentrations of Cu in the soil were observed in treated wastewater-irrigated soil, except under monocultured maize, in which the Cu concentration was higher in groundwater-irrigated soil (Figure 2b). Significant differences were observed in Pb and Cd concentration in the soil with a higher concentration in wastewater-irrigated soil compared to groundwater (Figure 2c,d). The concentration of Pb was higher in treated wastewater-irrigated uncultivated soil (Figure 2c), similarly to Cd, whose concentration in soil was also higher in treated wastewater-irrigated uncultivated soil (Figure 2d). This study showed that maize/soybean intercropping reduces Pb and Cd concentrations in soil, while it increases the concentration of Cu.

**Figure 3:**
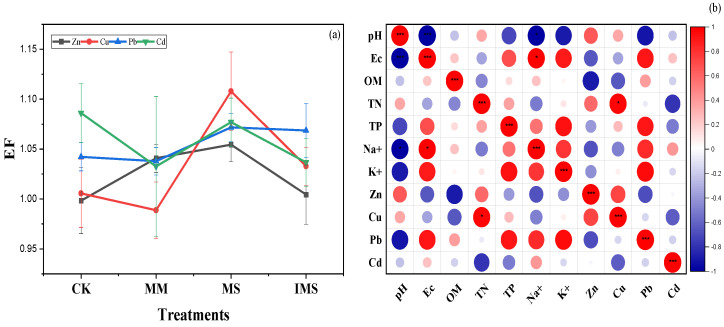
Enrichment factor for Zn, Cu, Pb, and Cd (**a**), and correlation between soil chemical properties and heavy metals enrichment factor (**b**). Note: CK: irrigated and uncultivated soil; MM: monocultured maize soil; MS: monocultured soybean soil; IMS: intercropped maize/soybean soil. All data are represented as means ± standard errors (n = 6) (* *p* < 0.05, *** *p* < 0.0001).

Significant differences were found in the Cd enrichment factor (EF) depending on used crop and planting patterns (Figure 3a). The highest enrichment factor was observed for Cd in treated wastewater-irrigated uncultivated soil, followed by Cu under soybean monocropping system. Significant differences were observed in Zn and Pb enrichment factors depending on the planting patterns (Figure 3a). Similarities were noted in Zn and Cu enrichment factors under uncultivated, monocultured maize, and intercropped maize/soybean soil. The Pearson correlation between soil chemical properties and heavy metals enrichment factors showed that soil chemical properties were not related to heavy metals enrichment factor (Figure 3b).

### 3.5. Heavy Metals Accumulation in Soil and Mobility in Plants

**Figure 4:**
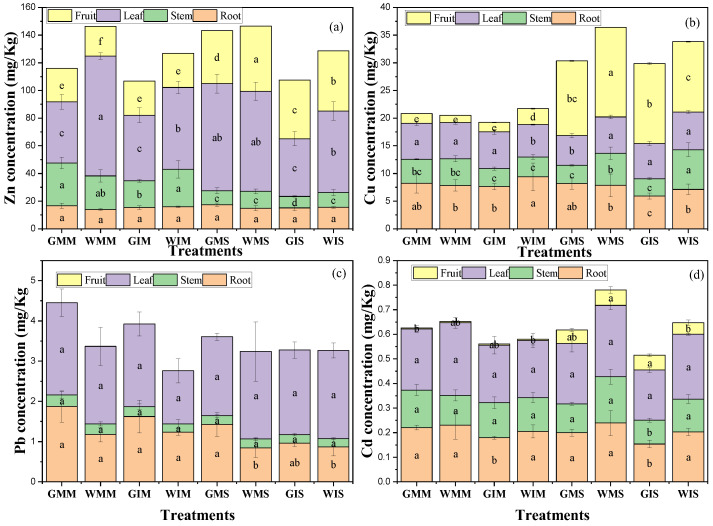
Heavy metals’ mobility in various crops’ organs under intercropping of maize and soybean. Zn concentration (**a**); Cu concentration (**b**); Pb concentration (Pb was not detected in fruit) (**c**); Cd concentration (**d**). All data are represented as means ± standard errors (n = 6). Different letters indicate statistically significant differences between treatments at *p* < 0.05.

Significant differences were observed in heavy metals’ migration from soil to various plant organs depending on the water treatment and planting pattern (Figure 4). The highest concentrations of heavy metals were found in leaves and roots. The concentration of Zn, Pb, and Cd was significantly higher in leaves (Figure 4a,c,d), contrastingly to Cu which showed a great affinity with the roots system (Figure 4b). The concentrations of heavy metals in the stem were not significantly altered compared with roots and leaves, except for Cu and Cd in which a slight increase was noted in plant stems. In addition, this study showed that heavy metals’ migration in plant parts was significantly affected by plant organs’ affinity towards the heavy metals.

The grains analysis showed that Zn was higher in wastewater-irrigated maize grains both in mono- and intercropping systems, while Cu concentrations were higher in intercropped maize grain in wastewater-irrigated soil (Figure 4a,b). The results showed that the concentrations of Zn, Cu, and Cd were higher in monocultured soybean grain in treated wastewater irrigation compared with groundwater. Contrastingly to the monocropping system, heavy metals’ concentration was higher in intercropped soybean irrigated with groundwater (Figure 4c). Treated wastewater irrigation increased crop growth and yield in mono- and intercropping systems without high concentrations of toxic heavy metals in grains with Cd in the acceptable range for safe consumption (GB 2762-2017), and Pb was not detected in maize and soybean grain in all treatments. This study showed that treated livestock wastewater could be considered as a serious alternative to reduce water scarcity in agricultural systems.

**Figure 5:**
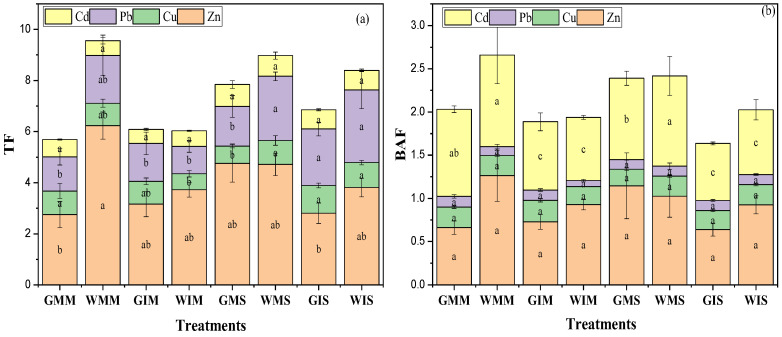
Translocation factor (**a**) and bioaccumulation (**b**) for Zn, Cu, Pb, and Cd in crops. All data are represented as means ± standard errors (n = 6). Different letters indicate statistically significant differences between treatments at *p* < 0.05.

Significant differences were found in heavy metals’ mobility between water treatments (Figure 5) and cropping patterns. The translocation factor values for Zn in treated wastewater irrigation were ranged between 6.24 mg/kg and 1.2 mg/kg; whereas, they were between 0.51 mg/kg and 0.49 mg/kg in groundwater irrigation. In addition, a higher Zn translocation factor was observed on monocropped maize in treated wastewater irrigation compared with in the intercropping system (Figure 5a). No significant difference was observed in the Pb and Cd translocation factors depending on the water treatment and planting pattern. The Cu translocation factor was higher in treated wastewater irrigation compared to groundwater. The intercropping system increased TF of soybean, contrastingly to maize where the TF was higher in monoculture except for Zn.

This study showed that water treatments and planting patterns have significantly impacted heavy metals bioaccumulation factor. The results revealed that treated wastewater and intercropping system increased Zn accumulation in maize cultivated soil. Similarities were observed for Cu, Pb where no significant differences were observed on bioaccumulation factor depending on water treatment and the type of culture (Figure 5b). Contrastingly in maize cultivated soil, Cd accumulation factor increased in groundwater-irrigated soil and under monocultured soybean. However, higher Cd bioaccumulation factor was observed in wastewater-irrigated soil and monocultured maize (Figure 5b).

### 3.6. Heavy Metal Transfer from Soil to Plants

**Figure 6:**
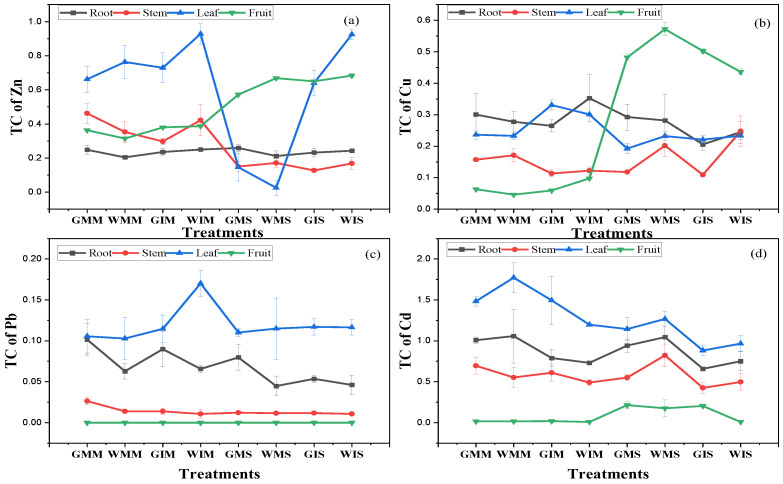
Variation in heavy metals’ transfer coefficient under different water treatments and planting patterns (Pb was not detected in fruit). All data are represented as means ± standard errors (n = 6). Different letters indicate statistically significant differences between treatments at *p* < 0.05.

Figure 6 shows the transfer coefficients (TCs) of Zn, Cu, Pb, and Cd concentrations calculated for each plant part. TC is a function for both soil and plant properties quantifying the relative differences in bioavailability of soil metal to plant. This study showed that the TCs for Zn, Cu and Pb were higher under intercropping system compared with monoculture (Figure 6a–c). A contrary situation was observed in the TC of Cd suggesting a higher retention of this metal in soil (Figure 6d). The TC of Pb was stable in stems contrastingly for roots and leaves in which significant variations were observed based on the water treatment and type of culture. This study suggests that intercropping system increase heavy metals TCs from soil to plant parts in groundwater and treated wastewater-irrigated soil excluding Cd.

## 4. Discussion

This study determined the effects of treated wastewater on plant growth and the migration of four heavy metals (Zn, Cu, Pb, and Cd) in soil–plant systems in mono- and intercropping systems. As previously suggested, treated wastewater irrigation increases crops’ growth and yield [5]. Our results are in line with [36] that an intercropping system increased plant growth and soil health (Table 2 and Table 4). This study revealed that heavy metal concentrations in aerial parts were significantly higher compared with the root, except for Cu in maize. Though heavy metal concentrations in plant parts were higher in treated wastewater and under the monocropping system, the transfer efficiency from soil to different plant parts was greater under the intercropping system compared with the monocropping system (Figure 6). In addition, Pb was not detected in grains in all the treatments, while the concentration of Cd was in an acceptable range for human consumption [37]. This study suggests that treated livestock wastewater could be considered as a great alternative without shadow effects to reduce the pressure of freshwater use in irrigation systems.

Crop interactions at the rhizosphere level can increase or hinder plant growth and nutrient uptake depending on the type of crop, irrigation treatment, and planting pattern [38]. Crop selection plays a significant role in these interactions and potential yield. In addition, treated wastewater irrigation has been characterized as a plant growth trigger, thus improving soil nutrient content. This study showed that livestock-treated wastewater irrigation significantly increased maize and soybean growth. Similar results were reported by ref. [39], suggesting that treated livestock wastewater improves soil quality and promotes plant growth. However, it is important to mention that these positive effects of treated wastewater on crop growth can be affected by the planting pattern. For instance, maize presents its optimal growth rate in monoculture compared with an intercropping system. This study showed that though treated wastewater irrigation increases plant growth, more significant positive impacts were observed on monocultured maize, contrastingly to soybean which showed significant positive effects in treated wastewater irrigation under mono- and intercropping systems compared with groundwater. In addition, intercropped soybean showed its highest growth rate in wastewater irrigation compared with groundwater. This situation could be explained by an abundant amount of nutrient and nitrogen increase by wastewater compared with groundwater irrigation, where the maize would use significant amounts of nitrogen and other nutrients. Alternatively, the intercropping system could increase nutrient availability with soybean’s great affinity with nitrogen [25].

This study revealed that treated wastewater increased crop leaves’ chlorophyll and nitrogen contents. This result is in line with ref. [40] that said treated wastewater irrigation increased crop physiological parameters. This study showed that treated wastewater could reduce the pressure of crop nutrient uptake from the soil and enhance plant physiological parameters under the intercropping system [41]. Soybean leaf chlorophyll and nitrogen contents increased under intercropping in groundwater irrigation compared with the monocropping system. It has been suggested that the intercropping system reduces the negative effects of trace elements and increases plant physiological characteristics including chlorophyll and carotenoids [25]. Moreover, intercropping promotes antioxidant enzyme activities, eliminating reactive oxygen species (ROS), and decreases the toxicity of trace elements on plants [25]. The improved leaf chlorophyll and nitrogen content might also be due to positive rhizosphere changes under intercropping. Previous studies reported that soybean could fix and mobilize atmospheric nitrogen in the rhizosphere [42,43]. As one of the major nutrients, N nutrition is vital for plant growth and optimum yields, especially for maize. Abundant nutrients promote microorganisms’ growth under intercropping which produces growth-promoting substances for crops [28]. However, maize and soybean showed a fluctuant trend under intercropping between groundwater and wastewater but a consistent increase in groundwater irrigation for soybeans under the intercropping system.

Heavy metals’ accumulation was higher in treated wastewater and uncultivated soil (Figure 5b). This study showed the significant role played by the intercropping system on heavy metals’ migration in soil–plant systems (Figure 2). The decrease in BAF in cultivated soil is due to the ability of soybeans to accumulate significant amounts of Cd (Figure 2). Zn, Pb, and Cd concentration was higher in leaves compared with roots and stems, contrastingly to Cu which showed a higher concentration in the roots. This study showed that intercropping affected heavy metals’ migration in soil–plant systems. However, these effects depend on heavy metals’ affinity towards plant parts. For instance, a higher concentration of Cu was observed in the roots contrastingly to Zn, Pb, and Cd. The concentration of Pb in stems was not significantly higher compared with that in leaves and roots. Planting pattern affects heavy metals’ migration from the soil to different plant parts. For instance, this study shows that heavy metals’ concentration in roots was higher in monocultured compared with the intercropping system and the transfer efficiency from roots to leaves was higher in the intercropping system compared with the monocropping system. The results of our experiment are in line with ref. [44], in which the concentration of heavy metals in the shoot cells of *A. alpina* was 18.8% higher in the intercropping system compared with the monocropping system irrigated with livestock-treated wastewater. In addition, a study conducted with a soil Cd concentration of 5 mg/kg revealed that intercropping with four Cd-accumulator plants used in floriculture reduced the Cd levels in grape plants [45]. Intercropping enhances the “communication pathways” between two plant species through the roots and increases the release of organic acids and other root exudates which could facilitate heavy metals’ movements in different plant parts. Under the intercropping system, a substantial change in the quantity and structure of plant roots in the soil may change the fractions of heavy metals and their distribution throughout the plants [44]. In addition, it was found that the Cd concentration in the grape was not significantly affected when the grape plant was intercropped with *Solanum nigrum* [46]. Our research demonstrated that leaves of intercropped maize and soybean accumulated more heavy metals than when monocropped except for Cu. Similar results were reported by ref. [44] that the accumulated Cd in the intercropped *V. faba* was greater than that of the monoculture.

BAF and TF were used to determine the metal accumulation characteristics of plants [18]. Heavy metals’ concentration in roots under the intercropping system was not significantly higher compared with that in roots in monocultured, suggesting that the intercropping system increases heavy metals’ transfer from soil to aerial plant parts. This situation might be due to maize/soybean interactions in the rhizosphere level affecting heavy metals’ transfer.

Continuous use of roughly treated wastewater in irrigation systems has been characterized as a major source of heavy metals’ soil contamination [17], which is a crucial challenge for sustainable agriculture. Hence, providing solutions for the safe utilization of treated wastewater for crop irrigation has become a mainstream objective to reduce the excess of freshwater use and increase food production. It has been suggested that intercropping of *S. alfredii* and oilseed rape reduced Cd concentration in wastewater-irrigated soil and simultaneously increased the production [25]. This study is in line with previous studies showing that intercropping promotes crop growth and increases heavy metals’ transport from soil to various plant organs [18,25,46]. Thus, the intercropping system increases crop growth and productivity and reduces the risk of soil’s heavy metals accumulation under treated wastewater irrigation.

## 5. Conclusions

The application of treated wastewater for irrigation purposes remains a major alternative to water scarcity in agricultural systems. This study revealed that treated wastewater irrigation and intercropping systems improve soil health and increase crop growth, yield, and grain nutrient content while reducing the concentration of toxic heavy metals under the intercropping system. In addition, the intercropping system enhances heavy metals’ transport in soil–plant systems, reducing potential soil heavy metal pollution. The concentration of heavy metals in grains was also under permissible limits for human consumption. The results showed comprehensive benefits of wastewater reuse in irrigation based on soil health, crop growth, and yield. This study demonstrated that treated livestock wastewater and the intercropping system could be proposed as a feasible long-term solution to water scarcity and to obtain economic returns and safe products for farmers. However, long-term research on heavy metals’ migration in soil–plant systems in an open field under a maize/soybean intercropping system needs further studies.

## Figures and Tables

**Table 1:** Chemical characteristics of soil, groundwater, and livestock wastewater.

Properties	Soil	Groundwater	Livestock Wastewater
pH	8.54	7.46	8.23
EC	1.330 mS/cm	1.088 mS/cm	26 mS/cm
OM	1.55 mg/kg		
COD		-	7818 mg/L
TN		0.433 mg/L	1675 mg/L
TP	0.6 mg/kg	0.037 mg/L	175.5 mg/L
Exchangeable potassium	108 mg/kg		
Water-soluble K^+^		1.7 mg/L	1022 mg/L
Water-soluble Na^+^		1.5 mg/L	690 mg/L
Cd	0.22 mg/kg	-	0.002 mg/L
Pb	16.08 mg/kg	0.2 mg/L	0.015 mg/L
Zn	61.35 mg/kg	0.102 mg/L	12.616 mg/L
Cu	24.13 mg/kg	0.004 mg/L	4.958 mg/L
Ca	10.66 mg/kg	82.58 mg/L	74.311 mg/L
Mg	1.95 mg/kg	61.3 mg/L	14.180 mg/L
Cr		0.013 mg/L	0.139 mg/L
Mn		0.105 mg/L	1.445 mg/L
Ni		0.001 mg/L	0.274 mg/L

**Table 2:** Description of the experimental treatments.

Treatment	Water Type	Planting Pattern	Crop
GMM	Groundwater (G)	Monocropping (M)	Maize (M)
WMM	Wastewater (W)	Monocropping (M)	Maize (M)
GMS	Groundwater (G)	Monocropping (M)	Soybean (S)
WMS	Wastewater (W)	Monocropping (M)	Soybean (S)
GIMS	Groundwater (G)	Intercropping (I)	Maize (M) + Soybean (S)
WIMS	Wastewater (W)	Intercropping (I)	Maize (M) + Soybean (S)
GCK	Groundwater (G)	None	None
WCK	Wastewater (W)	None	None

**Table 3:** (**a**) Maize growth parameters under different water treatments and planting patterns. (**b**) Soybean growth parameters under different water treatments and planting patterns. Different letters indicate statistically significant differences between treatments at *p* < 0.05.

**(a)**						
**Parameters**	**PP**	**WT**		**Two-Way ANOVA**		
		**G**	**W**	**WT**	**PP**	**WT × PP**
PH (cm)	MM	149.20 ± 1.08 b	156.60 ± 0.36 a	**	ns	***
	IM	156.80 ± 0.53 a	143.60 ± 0.91 b			
STD (m^2^)	MM	11.64 ± 0.62 c	13.28 ± 0.69 a	**	*	**
	IM	13.63 ± 0.41 a	12.55 ± 0.27 b			
SDW (g)	MM	57.78 ± 2.09 c	55.49 ± 3.40 d	***	**	***
	IM	75.38 ± 6.08 a	72.63 ± 5.16 b			
RDW (g)	MM	21.98 ± 1.35 ab	21.19 ± 0.86 ab	***	***	***
	IM	25.41 ± 2.08 a	23.20 ± 3.94 a			
LA (cm^2^)	MM	191.85 ± 2.91 b	190.88 ± 2.20 b	***	**	***
	IM	225.30 ± 1.94 ab	242.09 ± 3.07 a			
LWR (cm^2^g^−1^)	MM	2.64 ± 0.06 b	2.62 ± 0.15 b	***	***	***
	IM	2.99 ± 0.26 ab	3.27 ± 0.42 a			
**(b)**						
**Parameters**	**PP**	**WT**		**Two-Way ANOVA**		
		**G**	**W**	**WT**	**PP**	**WT × PP**
PH (cm)	MS	73.80 ± 0.50 b	79.60 ± 1.93 ab	*	ns	***
	IS	67.40 ± 1.46 c	81.20 ± 1.54 a			
STD (cm^2^)	MS	3.93 ± 0.10 ab	4.76 ± 0.13 a	**	ns	ns
	IS	3.07 ± 0.20 b	3.93 ± 0.05 ab			
SDW (g)	MS	27.14 ± 2.24 b	33.51 ± 2.44 a	***	**	ns
	IS	14.24 ± 0.71 c	17.54 ± 1.93 c			
RDW (g)	MS	16.11 ± 0.67 b	20.25 ± 0.18 a	***	***	***
	IS	11.084 ± 1.03 c	18.76 ± 1.19 ab			
LA (cm^2^)	MS	17.56 ± 1.26 b	21.31 ± 0.37 a	***	***	***
	IS	18.94 ± 2.10 b	21.70 ± 0.50 a			
LWR (cm^2^g^−1^)	MS	1.68 ± 0.01 a	1.65 ± 0.11 a	***	**	***
	IS	1.41 ± 0.05 b	1.48 ± 0.04 b			

Note: G = groundwater; W = wastewater; PH = plant height; STD = stem diameter; SDW = shoot dry weight; RDW = root dry weight; LA = leaf area; LWR; leaf weight ratio; PP: planting pattern; WT: water treatment; MM: monocropped maize; IM: intercropped maize; MS: monocropped soybean; IS: intercropped soybean. (ns: non-significant, * *p* < 0.05, ** *p* < 0.01, *** *p* < 0.001).

**Table 4:** (**a**) Effects of treated wastewater and planting patterns on yield of maize. (**b**) Effects of treated wastewater and planting patterns on yield of soybean.

(**a**)								
**Variables**	**Maize Kernel Weight per Plant (g)**		**Number of Seeds per Plant**		**Number of Seeds per Pots**		**100 Seeds Weight (g)**	
	**MM**	**IM**	**MM**	**IM**	**MM**	**IM**	**MM**	**IM**
G	55.63 ± 1.6 a	55.47 ± 5.3 b	133.67 ± 10.2 a	143.33 ± 15.7 b	267.33 ± 30.3 a	136 ± 23.1 b	29.37 ± 1.8 a	25 ± 1.5 a
W	48.82 ± 3.9 b	60.59 ± 5.6 a	129 ± 12.4 b	150.67 ± 16.5 a	258 ± 24.8 b	270.67 ± 23.7 a	26.27 ± 1.5 a	26.46 ± 0.3 a
WT	ns ns ns		ns ns ns		ns ns ns		ns ns ns	
PP								
WT*PP								
(**b**)								
**Variables**	**Number of Pods per Plant**		**Number of Seeds per Plant**		**Number of Seeds per Pots**		**100 Seeds Weight (g)**	
	**MS**	**IS**	**MS**	**IS**	**MS**	**IS**	**MS**	**IS**
G	22 ± 1 b	24 ± 0.6 a	57.67 ± 5.7 b	31 ± 4.1 a	120.67 ± 5.4 b	60.67 ± 5.2 a	17.47 ± 0.5 b	14.65 ± 1.3 b
W	33 ± 3.6 a	21.33 ± 0.9 b	64 ± 5.1 a	28 ± 1.5 b	128.67 ± 3.8 a	55.667 ± 3.1 b	20.01 ± 1 a	17.55 ± 1.2 a
WT	ns * **		ns ** ns		ns ns ns		* * ns	
PP								
WT*PP								

MM: monocultured maize, IM: intercropped maize. MS: monocultured soybean, IS: intercropped soybean. WT: water treatment. PP: planting pattern. Different lowercase letters in the same column indicate a significant difference between treatments at 0.05 level (ns: non-significant, * *p* < 0.05, ** *p* < 0.01).

**Table 5:** Soil chemical properties for each treatment.

Treatments	pH	EC (µS/cm)	OM (%)	TN (mg/g)	TP (mg/g)	Water-Soluble Na^+^ (mg/g)	Water-Soluble K^+^ (mg/g)
GMM	8.54 ± 0.02 a	281.33 ± 6.83 de	2.14 ± 0.04 a	1.06 ± 0.006 c	0.71 ± 0.03 c	0.02 ± 0.001 b	0.01 ± 0.001 e
WMM	8.52 ± 0.02 a	297 ± 4.90 d	2.12 ± 0.04 a	1.07 ± 0.040 c	0.80 ± 0.16 b	0.03 ± 0.002 ab	0.02 ± 0.002 d
GMS	8.54 ± 0.01 a	274.66 ± 3.43e	2.26 ± 0.02 a	1.10 ± 0.030 b	0.86 ± 0.31 ab	0.01 ± 0.001 c	0.04 ± 0.002 c
WMS	8.51 ± 0.02 a	286.97 ± 3.23de	2.10 ± 0.07 a	1.12 ± 0.033 b	0.85 ± 0.16 ab	0.03 ± 0.002 ab	0.05 ± 0.002 bc
GIMS	8.54 ± 0.02 a	277.98 ± 4.66e	2.01 ± 0.15 b	1.08 ± 0.016 c	0.80 ± 0.18 b	0.02 ± 0.001 b	0.02 ± 0.002 d
WIMS	8.39 ± 0.07 ab	363.68 ± 5.57c	2.39 ± 0.15 a	1.12 ± 0.032 b	0.84 ± 0.18 ab	0.03 ± 0.001 ab	0.04 ± 0.002 c
GCK	8.39 ± 0.02 ab	536.5 ± 5.40b	1.94 ± 0.03 b	1.07 ± 0.04 c	0.91 ± 0.04 a	0.04 ± 0.001 a	0.06 ± 0.005 b
WCK	8.08 ± 0.04 b	1013.6 ± 9.36a	1.87 ± 0.07 b	1.30 ± 0.030 a	0.80 ± 0.05 b	0.10 ± 0.021 c	0.14 ± 0.010 a
WT	ns	***	ns	***	***	***	***
PP	ns	***	*	**	***	***	***
WT*PP	ns	***	ns	***	***	***	***

EC: electrical conductivity; TN: total nitrogen; OM: organic matter; TP: total phosphorus. WT: Water treatments. PP: Planting patterns. All data are represented as means ± standard errors (n = 6); different lowercase letters in the same column mean a significant difference at the 0.05 level. (ns: non-significant, * *p* < 0.05, ** *p* < 0.01, *** *p* < 0.001).

## Data Availability

Not applicable.
